# Backyard Raccoon Latrines and Risk for *Baylisascaris procyonis* Transmission to Humans

**DOI:** 10.3201/eid1509.090128

**Published:** 2009-09

**Authors:** L. Kristen Page, Chris Anchor, Ellen Luy, Sarah Kron, Grace Larson, Lauren Madsen, Kenneth Kellner, Timothy J. Smyser

**Affiliations:** Wheaton College, Wheaton, Illinois, USA (L.K. Page, E. Luy, S. Kron, G. Larson, L. Madsen, K. Kellner); Wildlife Division of the Forest Preserve District of Cook County, Elgin, Illinois, USA (C. Anchor); Purdue University, West Lafayette, Indiana, USA (T.J. Smyser)

**Keywords:** Baylisascaris procyonis, prevention, zoonoses, raccoons, letter

**To the Editor:** Raccoons (*Procyon lotor*) are abundant in urban environments and carry a variety of diseases that threaten domestic animals (*1*) and humans (2,*3*). A ubiquitous parasite of raccoons, *Baylisascaris procyonis* causes a widely recognized emerging zoonosis, baylisascariasis (*3*). Although only 14 human cases of severe *B. procyonis* encephalitis have been reported over 30 years (*4*), prevention is still a priority for public health and wildlife officials because of the seriousness of the resulting neurologic disease (*5*).

Raccoons prefer to defecate at latrines they create. Infected animals shed ≈20,000 eggs/g of feces (*3*), so latrines serve as the foci of parasite transmission (*6*). When latrines occur in close proximity to humans, the risk for zoonotic transmission increases (*2*). Because *B. procyonis* are transmitted by the fecal–oral route, young children have the greatest risk for zoonotic infection because of their tendency to put objects into their mouths (*1*,*2*). Many human cases have occurred in environments where latrines were near children’s play areas. Our objective was to determine which factors encourage raccoons to create latrines in human habitats. This information will allow public health officials and wildlife managers to develop strategies to educate the public and to ultimately prevent zoonotic transmission.

We surveyed 119 backyards for raccoon latrines in the suburbs of Chicago, Illinois, USA, near the Ned Brown Forest Preserve (n = 38; 42°01′55.05′′N, 88°00′00.62′′W, Cook County) and Lincoln Marsh (n = 81; 41°51′4.54′′N, 88°5′39.019′′W, DuPage County). Yards were selected on the basis of proximity to forest preserves and willingness of homeowners to participate in the study. We located latrines by systematically searching yards, giving special attention to horizontal substrates, such as piles of wood and the bases of large trees (*6*). We removed all fecal material to test for *B. procyonis* and stored it in plastic bags at –20^o^C until analysis. Composite samples that were at least 2 g underwent fecal flotation in Sheather solution (*7*) (at least 1 g of every fecal deposit at a latrine) (n *=*131). We identified *B. procyonis* eggs by microscopic examination on the basis of their size and morphologic appearance (*2*). Multiple slides were examined for ≈10% of the samples (randomly selected) to validate our results. Prevalence was considered the proportion of positive samples from all sampled yards.

Each yard was additionally surveyed for potential latrine substrates (*8*) and factors believed to attract or deter raccoons. The distance of each yard from the nearest forested habitat was calculated by using ArcGIS 9.0 (Geographic Information Systems, Redlands, CA, USA). We used homogeneity tests to identify differences in the proportion of yards with latrines present and to compare the prevalence of *B. procyonis* between study areas*.* Logistic regression and odds ratios were used to evaluate a main effect model composed of 10 yard attributes, including the presence of a pet, birdfeeders, garbage cans, and sandboxes, and to evaluate a simplified model in which attributes were combined to reflect the presence of food and latrine substrates, such as pet food, birdfeed, garbage and piles of wood or logs, respectively.

Latrines occurred in 61/119 yards (51%; 95% confidence interval [CI] 0.42%–0.60%). There was no significant difference in the proportion of backyards with latrines in proximity to Ned Brown (23/38, 82%) and Lincoln Marsh (38/81, 46%). The number of latrines per backyard ranged from 1 to 6 (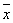 = 2.15). *B*. *procyonis* eggs were found at 14/61 latrines sampled (23%; 95% CI 12%–34%), and no significant difference in prevalence was found between the Ned Brown (6/23, 26%; 95% CI 8%–44%) and Lincoln Marsh areas (8/38, 21%; 95% CI 8%–34%).

Evaluation of the main effect model identified a decreasing probability of latrine occurrence with increasing distance from the nearest forested area and the presence of an outdoor pet, although these relationships were only marginally significant (p = 0.07 and 0.08, respectively). No other variables were closely associated with the presence of raccoon latrines (p>0.20). When evaluated alone, distance from the forest preserve was significantly related to latrine occurrence (p = 0.03); probability decreased with increasing distance. Evaluation of the simplified model identified a weakly positive association with the presence of a food source (p = 0.09) and no association with the presence of latrine substrate (p = 0.35). Although the findings were not statistically significant, raccoon latrines did appear to be associated with the availability of a food source such as bird feed (odds ratio [OR] 1.9, 95% CI 0.9–4.1); the presence of an outdoor pet (OR 0.27, 95% CI 0.06–1.2) and increasing distance from the nearest forested area reduced the likelihood of latrines. No other variables were associated with the presence of raccoon latrines; however, low statistical power may have precluded adequate assessment.

Our results suggest that when humans live close to protected forests or natural areas, they are more likely to attract raccoons into their yards. In addition, anthropogenic food sources such as pet food, garbage, and bird feed may increase the likelihood that a raccoon will create a latrine, and the presence of outdoor pets appears to be a deterrent. In areas of high raccoon density, these attractants should be removed. Homeowners with small children should remove latrines as quickly as they are discovered (*2*). The risk of children acquiring potentially fatal baylisascariasis can be reduced if parents understand how to reduce the likelihood that children will come into contact with raccoon latrines.
